# Impact of subinhibitory concentrations of metronidazole on proteome of *Clostridioides difficile* strains with different levels of susceptibility

**DOI:** 10.1371/journal.pone.0241903

**Published:** 2020-11-09

**Authors:** Tri-Hanh-Dung Doan, Stéphanie Yen-Nicolaÿ, Marie-Françoise Bernet-Camard, Isabelle Martin-Verstraete, Séverine Péchiné

**Affiliations:** 1 Université Paris-Saclay, INRAE, AgroParisTech, Micalis Institute, Jouy-en-Josas, France; 2 Université Paris-Saclay, UMS « Ingénierie et Plateformes au Service de l'Innovation Thérapeutique », Proteomic Facility, Châtenay-Malabry, France; 3 Laboratoire Pathogenèse des Bactéries Anaérobies, Institut Pasteur, Université de Paris, Paris, France; 4 Institut Universitaire de France, Paris, France; Emory University School of Medicine, UNITED STATES

## Abstract

*Clostridioides difficile* is responsible for various intestinal symptoms from mild diarrhea to severe pseudomembranous colitis and is the primary cause of antibiotic-associated diarrhea in adults. Metronidazole was the first-line treatment for mild to moderate *C*. *difficile* infections for 30 years. However, clinical failure and recurrence rates of metronidazole is superior to oral vancomycin and metronidazole is now recommended only as an alternative to vancomycin or fidaxomicin, for an initial non-severe infection. The mechanisms of treatment failure and infection recurrence remain unclear. Given the poor fecal concentrations of metronidazole, the bacteria may be exposed to subinhibitory concentrations of metronidazole and develop adaptation strategy, which is likely to be the origin of an increase in treatment failures. In this study, a proteomic approach was used to analyze changes in the proteome of two strains with different levels of susceptibility to metronidazole in the presence of subinhibitory concentrations of this antibiotic. The two strains were grown to stationary phase: CD17-146, a clinical *C*. *difficile* isolate with reduced susceptibility to metronidazole, and VPI 10463, a metronidazole susceptible strain. Our study revealed that, whatever the strain, subinhibitory concentrations of metronidazole modified the amount of proteins involved in protein biosynthesis, glycolysis, and protection against stress induced by metronidazole, as well as in DNA repair. Several proteins involved in stress response are known to be synthesized under the control of Sigma factor B, which suggests a close link between Sigma factor B and metronidazole. Interestingly, impact of metronidazole on protein production for VPI 10463 strain differed from CD17-146 strain, for which the amount of two proteins involved in biofilm formation of CD17-146 were modified by metronidazole.

## Introduction

*Clostridioides difficile* is a Gram-positive, spore forming and obligate anaerobic bacterium responsible for post-antibiotic diarrhea with a spectrum of clinical signs, ranging from self-limiting diarrhea to life-threatening pseudomembranous colitis. The *C*. *difficile* infection (CDI) has become the most common cause of healthcare associated infections in the United States, and the most frequent hospital-acquired intestinal infection in Europe and worldwide [1,2].

The usual treatment for CDI requires the use of antibiotics with activities against *C*. *difficile*. Vancomycin, metronidazole (MTZ) and fidaxomicin were drugs of choice. For decades, MTZ, which has selective activity against anaerobic or microaerophilic bacteria [3] was widely used as first-line therapy because of its lower cost. Vancomycin therapy is superior to MTZ for clinical cure [4] but its recurrence rate is still high, approximately 25% [5,6]. Fidaxomicin, with a narrow spectrum, appears similar to vancomycin for the clinical response at the end of CDI therapy but has a reduced risk of recurrences and a lower impact on the intestinal microbiota [7]. However, a broad use of fidaxomicin is limited by its high cost [7]. The recommendation for use of MTZ, nevertheless, has recently changed. MTZ was widely used as first-line antibiotic in the treatment of mild-to-moderate CDI. Since 2018, several studies reported the emergence and spread of *C*. *difficile* clinical isolates resistant to MTZ [8], as well as treatment failure and high recurrent rate (20–25%) post MTZ therapy [4]. MTZ is now recommended only as an alternative to vancomycin or fidaxomicin, for an initial non-severe infection [1]. It is of increasing importance to understand in more details the mechanisms of adaptation and resistance of this pathogen to MTZ.

Despite decades of research, the metabolism of MTZ and the stress response to this antibiotic in bacteria were not definitively characterized. Being a prodrug, MTZ is inactive until taken up and reduced under low oxygen tension by pyruvate-ferredoxin oxidoreductase (PFOR) in concert with ferredoxin. PFOR and ferredoxin were proposed to be the only couple in anaerobic bacteria with a sufficiently low redox potential to reductively activate metronidazole [9]. Later it was discovered that effectors with very negative midpoint redox potentials, including purified ferredoxin, flavodoxin [10] and hydrogenase [9] from *C*. *pasteurianum* were able to reductively activate metronidazole directly. Reduction of MTZ is proposed to form nitroso free radical and hydroxylamine, which exert cytotoxicity by direct DNA damage. For survival, bacteria can reduce MTZ uptake, decrease the rate of MTZ activation or increase DNA repair [11].

In *Bacteroides fragilis*, several mechanisms of resistance to MTZ have been described such as the presence of 5-nitroimidazole reductase (Nim) [12–16], an altered expression of redox-active proteins [17] and DNA repair RecA protein [18]. Mechanisms for MTZ resistance have also been described in *Helicobacter pylori* including inactivation of *rdxA* (NADPH dependent nitroreductase) and *frxA* (NAD(P)H flavin oxidoreductase) genes [19–21], alterations in activity of non-*rdxA* encoded nitroreductases [22], mutations within the gene encoding the ferric uptake regulator (*fur*) [23,24], overexpression of *recA* [25,26], and of the gene encoding the HefA bacterial efflux pump [27]. The MTZ resistance in *C*. *difficile* is due to multifactorial mechanisms not yet fully understood. Current data have suggested the possible involvement of alterations in several defined metabolic pathways, such as those implicated in the activity of nitroreductases, iron uptake, and DNA repair [28,29]. Interestingly, a recent study has shown the correlation between the resistance to MTZ (minimal inhibitory concentration MIC  =  8 μg.mL^-1^) of a PCR ribotype 020 strain and the presence of a plasmid, pCD-METRO [30]. Another study on chromosomal resistance to MTZ in *C*. *difficile* demonstrated that a first-step mechanism of low-level resistance is a truncation of the ferrous iron transporter, FeoB1. Higher-level resistance evolved from sequential acquisition of mutations to catalytic domains of pyruvate-ferredoxin/flavodoxin oxidoreductase (PFOR), a synonymous codon change to putative *xdh* (xanthine dehydrogenase; encoded by *CD630_31770*), and frameshift and point mutations that inactivated the iron-sulfur cluster regulator (*iscR*). However, resistance involving these genes was seen only in the *feoB1* deletion mutant and not in the isogenic parental strain [31].

When MTZ is administered orally, it is absorbed rapidly and almost completely in small intestine, with only 6% to 15% of the drug excreted in the stool. In asymptomatic patients, stool concentrations of MTZ were undetectable [32]. In symptomatic patient, stool concentrations of MTZ were detected with a mean concentration of 9.3 mg/g in watery stool and only 1.2 mg/g in formed stool. The poor fecal concentrations of MTZ might be insufficient to inhibit the growth of vegetative cells of *C*. *difficile* [33], promoting the development of adaptation and resistance mechanisms of *C*. *difficile*. Indeed, in a study on fecal samples to assess pathogen reduction of vancomycin and MTZ in CDI therapy, treatment with vancomycin consistently reduced the number of *C*. *difficile* to the limit of detection, while MTZ was associated with mean *C*. *difficile* counts of 3.5 to 4 log10 after 10 days of treatment [34]. In vitro, susceptibility to MTZ in *C*. *difficile* was shown to be heterogeneous, which means that growth in the presence of MTZ may select slow growing subclones with higher MICs of MTZ in a population with low MICs [35]. Moura et al. also found that following exposure to sub-MIC concentrations of MTZ, *C*. *difficile* PCR strains ribotype 001 and 010 exhibited a higher MIC [36]. In addition, a recent study reported that subinhibitory concentrations of MTZ may be a stress factor able to induce biofilm formation in *C*. *difficile* [37].

For all these reasons, continued growth in the presence of subinhibitory antibiotic levels is a crucial aspect of the current antibiotic resistance crisis and increased therapeutic failures. Recent studies have shown that these low antibiotic concentrations exert their effects on at least three different levels: as selectors of resistance (by enriching for pre-existing resistant bacteria and by selecting for de novo resistance); as generators of genetic and phenotypic variability (by increasing the rate of adaptive evolution, including resistance development); and as signaling molecules (influencing various physio-logical activities, including virulence, biofilm formation and gene expression) [38]. The idea that sub-MIC antibiotic concentrations can have a broad range of physiological and morphological effects on bacteria has been discussed since the very early days of clinical antibiotic use [39].

In order to elucidate the effect of MTZ on the proteome of *C*. *difficile* strains, we described proteomic adaptation of a *C*. *difficile* isolate, CD17-146, with reduced susceptibility to MTZ and VPI 10463, a MTZ susceptible strain in response to subinhibitory concentrations of the antibiotic. This proteomic study was done by Liquid Chromatography-Tandem Mass Spectrometry (LC-MS/MS) analysis after growth without MTZ or with increasing subinhibitory concentrations of MTZ (MIC/4 and MIC/2). We focus this analysis on the proteins involved in processes that have been already characterized to be associated with MTZ-resistance/adaptation in other bacteria. To complete this study, we selected some genes encoding interesting proteins for qRT-PCR analysis to examine their transcriptional control in response to MTZ.

## Materials and methods

### Bacterial strains

Two *C*. *difficile* strains VPI 10463 (ATCC 43255) and CD17-146 were used in this study. The CD17-146 isolate provided by the *C*. *difficile* French National Center in Saint Antoine hospital (Paris, France) was stored immediately after isolation at -80°C. This strain has been shown to be a non-toxigenic strain and belonging to the PCR ribotype 596 with reduced susceptibility to MTZ (minimum inhibitory concentration determined by ETEST® on solid agar was 2 μg.mL^-1^). We also used the 630Δ*erm* strain and the isogenic *sigB* mutant [40].

### Phenotypic tests and growth curve

Bacteria were grown at 37°C, under anaerobic conditions (90% N2, 5% CO2 and 5% H2), in fresh BHISG medium (Brain Heart infusion broth [Difco, USA] supplemented with 1.8% Glucose, 0.1% L-Cysteine and 0.5% yeast extract). BHISG media is widely used for growth and biofilm formation of *C*. *difficile* [37,41,42]. Broth dilution method was used to determine MIC defined as the lowest concentration of antibiotic preventing visible growth. For each strain, the MIC values of MTZ were evaluated by a method adapted from a previous study [43]. MIC experiments were done in duplicate by using two-fold serial dilution (from 0.125 to 2 μg.mL^-1^) with an inoculum of 10^5^ CFU per ml in BHISG. The MIC values were read after 24 h of incubation.

For the MTZ stress assays, 10 ml of the peptose medium (Pep-M) was inoculated. After an overnight culture at 37°C, the cultures of *C*. *difficile* strains were plated on Pep-M agar plates and a disk with 4 μg of MTZ was added. The diameter of the growth inhibition was measured after 24 h of incubation at 37°C [40]. Nine independent tests have been performed.

To examine the bacterial growth rate in the presence of MTZ, 100 μl of a pre-culture of *C*. *difficile* in BHISG with 10^8^ CFU.mL^-1^ were inoculated in 50 mL of BHISG containing 0.125, 0.25, or 0.5 μg.mL^-1^ of MTZ to obtain a final concentration of approximately 10^5^ CFU.mL^-1^ [44]. Growth curves were performed at 37°C in an anaerobic chamber. Growth rates were determined for two biological replicates by measuring optical density at 600 nm (OD_600nm_) and by numerations of viable cells on BHI agar supplemented with 3% of defibrinated horse blood. For each biological sample, bacterial enumerations have been performed in duplicate. Data was analyzed with Mann-Whitney U test with SPSS 20. Results are expressed as mean ± standard error of the mean (SEM).

### Proteomics analysis

#### Strains growth and extraction

Ten mL of BHISG were inoculated with 10^5^ CFU of either VPI 10463 or CD17-146, in absence or presence of MTZ at concentrations corresponding to 1/2 and 1/4 of the MIC value, different between the two strains. Cultures were incubated during 18 h at 37°C in anaerobic chamber to reach stationary phase to an OD_600nm_ of 1.2 for CD17-146 and 1.4 for VPI 10463. Cultures (0.1mL of tenfold serial dilutions) were collected for numeration of viable cells. Cultures were then pelleted (10 min, 3000 rpm, 4°C), and washed three times with sterile PBS. The extraction of proteins was performed as described in a previous study [45]. Pellets were resuspended in 200 μl of 0.062 M Tris (pH = 6.8), disrupted with 0.35g of glass beads (G4649, Sigma-Aldrich) and vigorously vortexed for 4 min, followed by centrifugation (20000 g, 20 min, 4°C). The supernatants were collected and stored at -20°C until used. Protein quantification was performed using a Bicinchoninic Acid (BCA) Protein Assay Kit, with bovine serum albumin (BSA) as standard (Pierce Protein Research Products, Thermo Fisher Scientific). A total of 10 μg of proteins from each sample were loaded into a 12% SDS-PAGE gel. A short migration was then performed allowing proteins to penetrate the gel. Gels were stained with Coomassie colloidal blue (EZblue, Sigma–Aldrich, France) to recover a 2 mm band containing all proteins.

*Protein in-gel digestion*. A reduction/alkylation step was performed with 10 mM of dithiothreitol (DTT) and 55 mM of iodoacetamide. Pieces of gels were rehydrated 1 h at 4°C in 12 ng/μL sequencing grade modified trypsin (Promega, France) solubilized in 25 mM NH_4_HCO_3_ and then digested overnight at 37°C. After tryptic digestion, peptides were extracted by incubating gel pieces in extraction solvent (0.1% formic acid/50% acetonitrile).

#### Liquid Chromatography-Tandem Mass Spectrometry (LC-MS/MS) analysis

Samples were analyzed on a nano-UPLC Ultimate 3000 RSLCnano (Thermo) on-line with a high-resolution mass spectrometer Q-Exactive (Thermo Scientific, France) using a nanoelectrospray interface in positive polarity mode, as described in a previous study [46]. For each sample, 4 μL of protein digest were loaded onto a Biosphere C18 Precolumn (0.1 × 20 mm, 100 Å, 5 μm; Nanoseparation) at 7.5 μL min^-1^ and desalted with 0.1% (v/v) formic acid (AF) and 2% (v/v) acetonitrile (ACN). After 3 min, the precolumn was connected to a Biosphere C18 Nanocolumn (0.075 × 300 mm, 100 Å, 3 μm; Nanoseparation).

Electrospray ionization was performed at 1.6 kV with an uncoated capillary probe. Buffers were 0.1% formic acid in water (A) and 100% ACN (B). Peptides were separated using a linear gradient from 5 to 30% buffer B for 75 min at 300 nL.min^−1^. One run took 95 min, including the regeneration step at 95% buffer B and the equilibration step at 95% buffer A. Peptide ions were analyzed using Xcalibur 4.01 Tune 2.7 (Thermo Electron) with the following data-dependent acquisition steps: (1) MS scan (mass-to-charge ratio (m/z) 350 to 1400, 70,000 resolution, profile mode), (2) MS/MS (17,500 resolution, normalized collision energy of 27, profile mode). Step 2 was repeated for the eight major ions detected in step (1). Dynamic exclusion was set to 50 s.

#### Data processing and statistical analysis

Peak lists were generated as mzXML files using MSConvert software (ProteoWizard 3.0). Mass spectrometric data was searched using X!TandemPipeline software version 0.2.38 developed by PAPPSO (Plateforme d’Analyse Protéomique de Paris Sud Ouest) facility [31]. Peptide searches were performed with the following parameters: enzymatic cleavage by trypsin digestion with one possible mis-cleavage, precursor mass tolerance of ±10 ppm and fragment mass tolerance of 0.5 Da, static modifications of carbamidomethylated cysteine and potential modification of oxidized Methionine. Uniprot KB/SwissProt *Clostridioides difficile* VPI10463 database and a homemade contaminant database (trypsin, keratin, etc.) were used. The genome sequence of VPI 10463 was used as reference for protein annotation. The identified proteins were filtered with a minimum of two different peptides required with a peptide *E-value* < 0.05, and a protein *E-value* (product of unique peptide *E values*) < 10^−3^. Combined analysis mode with all samples was performed and results collected from grouping proteins. Within each group, proteins with at least one specific peptide relatively to other members of the group were reported as subgroups. One subgroup represents one specific protein. The false discovery rates (FDR) obtained on peptide and protein identifications with a decoy databank were 0.05% and 0% respectively.

Label free comparative quantification of proteins was achieved through spectral counting (SC) following alignment by Mass Chroq version 2.2.12 [47]. Alignment process allows minimizing LCMS technical failure impact (such as inconsistent retention times). Spectral counting sums the number of spectra that has been identified to peptides belonging to a defined protein, to assess protein abundance (shared peptides are not taken into account).

Graphical representations were performed using MassChroqR package developed by PAPPSO team (R Studio version 1.0.153). After checking the normality and the homogeneity of the SC data, proteins showing less than 5 spectra in any of the injections were removed to focus on robust quantification. Proteins showing little variation in their number of spectra were removed. For each protein, the fold change is the ratio between the minimal and the maximal abundance values computed for the combination of factors ‘strain’ and ‘MTZ concentration’. In our study minimal fold change was set at 1.5, so only proteins showing variations between the minimal and the maximal abundance above 1.5 in their number of spectra were studied to focus on significant changes.

#### RNA extraction and quantitative RT-PCR analysis

*C*. *difficile* cultures were grown to an OD_600nm_ of 0.9 in BHISG, without or with MTZ at [MIC/4] or [MIC/2], RNAs were extracted using Trizol Reagent (Thermo Fisher Scientific) and RT-qPCR was realized as described by Batah et al. [48]. RNA quantification and quality were evaluated by a 2100 Bioanalyzer Agilent. cDNA was synthesized from 1 μg RNA using random primers and SuperScript™ III Reverse Transcriptase (Invitrogen) as described by the manufacturer. Real-time PCRs were done with 1 ng of cDNA template using SSo Advanced™ SYBR Green Supermix (Bio-Rad). The primers used are listed in S1 Table. Reactions were run on a CFX96 Real-time system (Bio-Rad) with the following cycling conditions: 30 s polymerase activation at 95 °C and 40 cycles at 95 °C for 5 s and 60 °C for 10 s. In order to verify the specificity of the real-time PCR reaction for each primer pair, an additional step from a start at 65 °C to 95 °C (0.5 °C/0.5 s) was performed to establish a melting curve. The *ccpA* gene was used as reference gene, as described in a previous study of Kint et al. [40]. Normalized relative quantities were calculated using the ΔΔCT method. Mann Whitney test with SPSS 20 software was used to evaluate whether the differences observed in the presence or absence of the antibiotic were significant for each strain. Differences were considered significant for fold change cut-offs of >2 and significance p-values of <0.05.

## Results and discussion

### Determination of Minimal Inhibitory Concentrations (MICs) of MTZ

The MIC of MTZ observed was 0.5 μg.mL^-1^ for VPI 10463 and 1 μg.mL^-1^ for CD17-146. However, the initial MIC of the CD17-146 strain was 2 μg.mL^-1^ as determined by ETEST®.

Such difference in MIC results can be explained by the use of different methods (E-TEST and broth dilution). Another possible factor that might have changed the susceptibility of CD17-146 to MTZ is the storage at -80°C. Indeed, the phenomenon of unstable MIC of MTZ for *C*. *difficile* has been previously described by Peláez et al [35]. In their study, the initial MICs determined for fresh isolates decreased after the isolates were thawed. The MIC/2 and MIC/4 were determined based on the results obtained by broth dilution method. According to epidemiological cut-off values of the European Committee on Antimicrobial Susceptibility Testing (ECOFF EUCAST2015), resistance to MTZ was defined as MIC> 2μg.mL^-1^. Most *C*. *difficile* susceptible strains have MIC ≤ 0.5 μg.mL^-1^ (https://mic.eucast.org/Eucast2/regShow.jsp?Id=21294). We considered CD17-146 as a strain with reduced susceptibility.

### Bacterial growth curves

**Fig 1:**
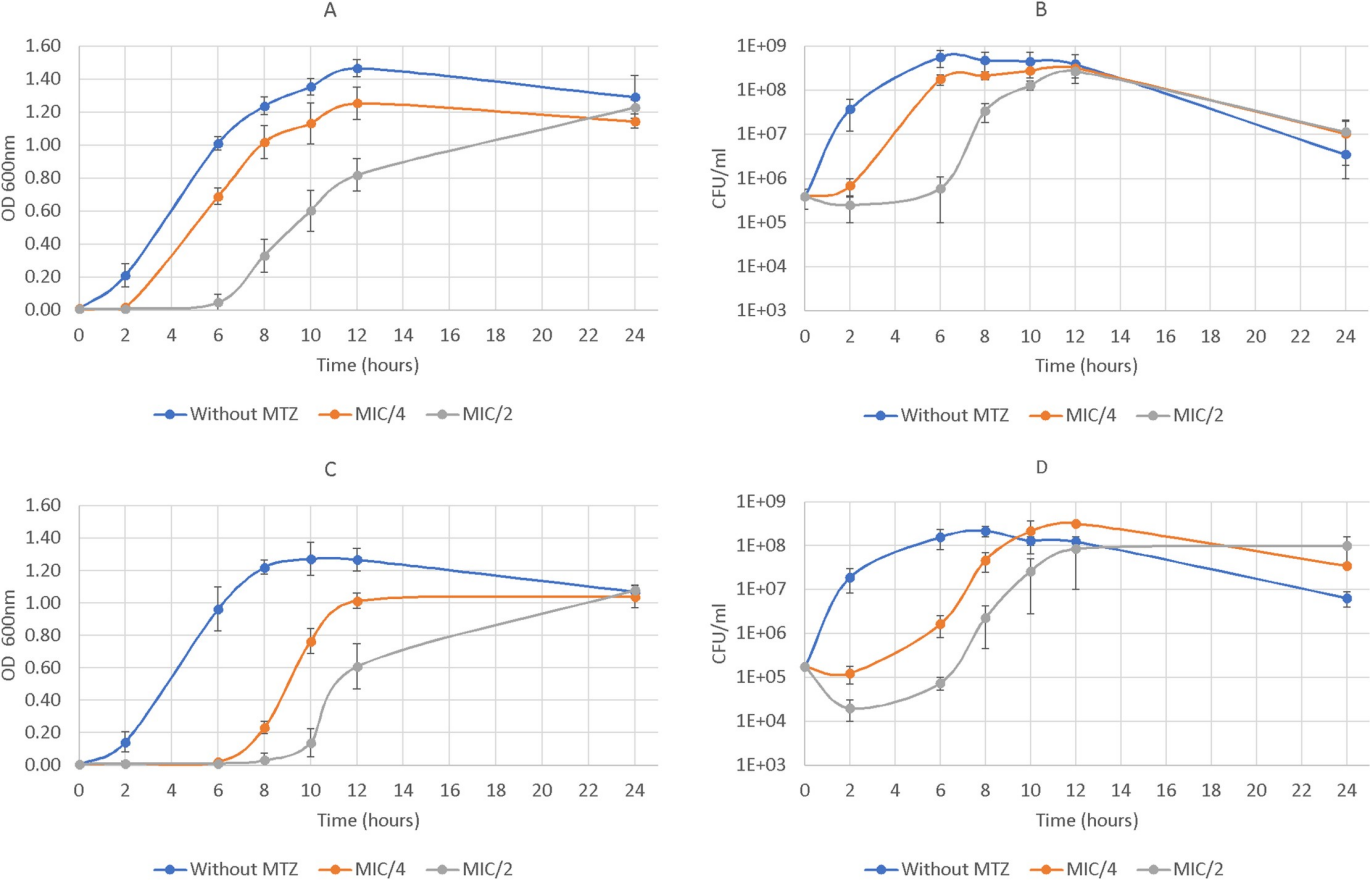
Effect of different concentrations of MTZ on growth of *C*. *difficile* VPI 10463 (A, B) and CD17-146 (C, D) strains. The bacteria were grown without MTZ (blue) or exposed to MIC/4 (orange) or MIC/2 (grey) of MTZ in BHISG medium. MIC for VPI 10463 and CD17-146 were 0.5 and 1 mg.mL^-1^, respectively. The growth rates were determined by measuring OD_600nm_ (A, C) and numeration of bacterial cells on BHI agar supplemented with 3% defibrinated horse blood (B, D). We observed that subinhibitory concentrations of MTZ had a direct inhibitory effect on the bacterial growth for both strains and CD17-146 grew more slowly than VPI 10463 in the presence of MTZ.

The dynamic of bacterial growth was monitored in liquid BHISG medium with a start inoculum of 10^5^ CFU.mL^-1^ of *C*. *difficile* without MTZ or supplemented with 0.125, 0.25, or 0.5 μg.mL^-1^ of MTZ. We found that subinhibitory concentrations of MTZ had a direct inhibitory effect on the bacterial growth for both strains (Fig 1). This effect was concentration dependent. For CD17-146 strain, the treatment with MTZ delayed the exponential phase for 6 h and 8 h at MIC/4 (0.25 μg.mL^-1^) and MIC/2 (0.5 μg.mL^-1^), respectively. However, for VPI 10463 lag phase was only prolonged for 2 and 6 h in the presence of MTZ at MIC/4 (0.125 μg.mL^-1^) and MIC/2 (0.25 μg.mL^-1^), respectively. The enumeration of bacterial viable cells confirmed the results observed by measuring OD_600nm_ and showed that lag phase for the CD17-146 strain was 3-fold and 1.3-fold longer than for VPI 10463 at MIC/4 and MIC/2 respectively, while the length of lag phase was similar between the two strains without MTZ. In addition, without MTZ, the growth rates of CD17-146 and VPI 10463 were similar (3.25 ± 0.57 h-1 and 3.17 ± 0.22 h-1, respectively). In contrast, in the presence of MTZ, strain CD17-146 seemed to grow more slowly than VPI 10463 at MIC/2. During the first 4 h of the exponential phase, the growth rates for VPI 10463 were 2.02 ± 0.27 h^-1^ at MIC/4 and 2.14 ± 0.52 h^-1^ at MIC/2, while for CD17-146, the growth rates were 1.74 ± 0.1 h^-1^ at MIC/4 and 1.21 ± 0.1 h^-1^ at MIC/2. However, there were no statistically significant differences between the growth rates of the two strains with or without MTZ. Slow-growing sub-populations with MTZ reduced susceptibility appeared after prolonged exposure of susceptible clinical isolates to MTZ in vitro. This selection process was sometimes reversible after passage in the absence of metronidazole; however, some slow-growing mutants converted to stable high-level resistance [15].

### Hierarchical clustering analysis showed divergence of behavior following increase of MTZ concentrations

To define more precisely the effects of MTZ on *C*. *difficile*, a proteomic approach was used to analyze proteome profiles of strains CD17-146 and VPI 10463. These two strains were grown 18 h to stationary phase in the presence of MTZ at subinhibitory concentrations. All the cultures of VPI 10463 strain had an OD_600nm_ of 1.4, the amounts of viable cells were 10^7^, 2 x 10^7^ and 9 x 10^7^ CFU for the culture without MTZ, with MTZ at MIC/4 or MIC/2, respectively. For CD17-146, the cultures had an OD_600nm_ of 1.2 with 2 x 10^7^, 4 x 10^7^ and 9 x 10^7^ CFU for the culture without MTZ, with MTZ at MIC/4 or MIC/2, respectively. These results suggested that the state of the cultures after 18 h of growth were approximately similar without or with the MTZ whatever the concentration.

Bacterial proteins in the cultures were extracted using glass beads. Tryptic digests of samples were analyzed by liquid chromatography combined with mass spectrometry. Using the X!tandem pipeline, we identified 1049 proteins with at least 2 peptides identified over all samples with a peptide false discovery rate (FDR) of 0.05%, and a protein FDR of 0%. Among them, we found 629 proteins whose production was significantly different with minimal fold change set at 1.5. A heatmap was created based on these proteins to map the behaviors of the two strains.

**Fig 2:**
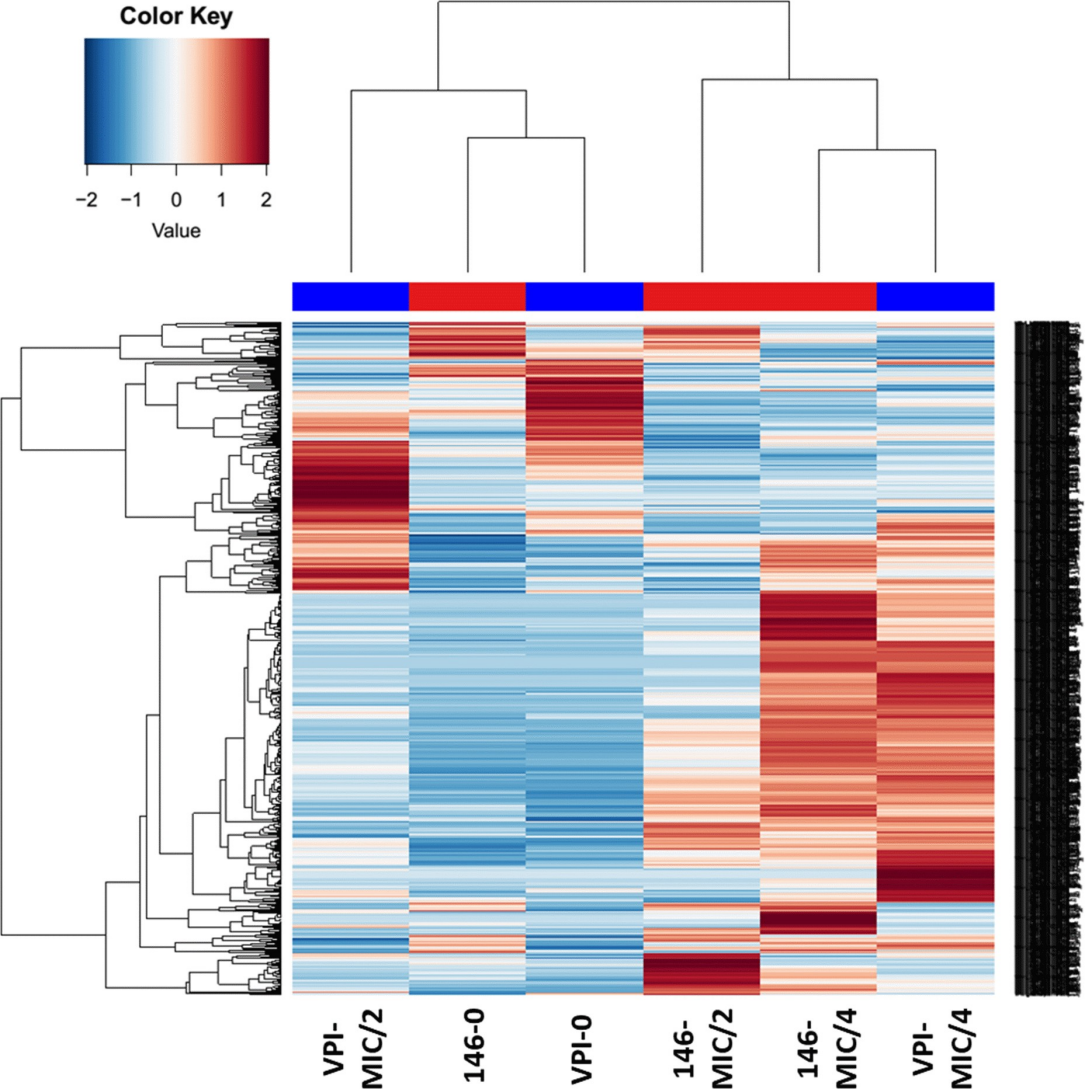
Hierarchical clustering analysis based on protein expressions of VPI 10463 and CD17-146 strains after culture in BHISG without MTZ, at MIC/4 and at MIC/2. Each column corresponds to a single sample: VPI-0 (VPI 10463, without MTZ), VPI-MIC/4 (VPI 10463, with MTZ at MIC/4), VPI-MIC/2 (VPI 10463, with MTZ at MIC/2), 146–0 (CD17-146, without MTZ), 146-MIC/4 (CD17-146, with MTZ at MIC/4), 146-MIC/2 (CD17-146, with MTZ at MIC/2). Each row corresponds to a single protein, blue indicating a reduced expression and red an increased expression. The proteome profiles of the strains CD17-146 and VPI 10463 were close without MTZ but reached opposite directions with MTZ at MIC/2, suggesting two types of MTZ stress response as stress gets more important.

Analysis revealed that the proteomes of the two strains CD17-146 and VPI 10463 in the absence of MTZ belonged to the same cluster of the heat map, as they showed more similarities among all profiles (Fig 2). The protein profiles of the two strains after growth at subinhibitory concentrations of MTZ were distinct from the non-treated controls indicating numerous significant changes at the level of production of various proteins when MTZ was added. Indeed, the proteomes of CD17-146 and VPI 10463 at MIC/4 were resolved in one major branch, while the proteomes of non-treated controls were resolved into a separate branch (Fig 2). Moreover, although protein production patterns were close for the two strains at MIC/4, the color change indicates a different behavior compared to the one in absence of MTZ. When reaching MIC/2, patterns took divergent directions. This suggested variable responses of the two strains to MTZ stress as stress gets more important.

To try to identify proteins involved in MTZ-stress response for each strain, we analyzed the proteins whose production was significantly different to focus on specific changes of proteins involved in cell envelope homeostasis, biofilm formation, protection against stress induced by MTZ and central metabolism.

### Cell envelope homeostasis and biofilm formation as a possible protection against MTZ

MTZ modified the production of *C*. *difficile* proteins involved in cell wall metabolism. For both strains, we observed an increased production of proteins implicated in the production of the disaccharide-pentapeptide required for peptidoglycan synthesis (MurB, MurC, MurD, MurF, MurG) or its association with a lipid carrier (UppS) and glutamate racemase (MurI) involved in synthesis of precursors of the disaccharide-pentapeptide. These proteins were detected in the presence of MTZ only, reaching the highest number of spectra at MIC/4. In contrast, the amount of MurA enzyme, which adds an enol-pyruvyl to UDP-N-acetylglucosamine, decreased slightly 1.8 and 1.4 –fold for VPI 10463 and CD17-146 respectively at both concentrations of MTZ Table 1.

**Table 1:** Differential expressions of proteins involved in cell envelope of VPI 10463 and CD17-146 *C*. *difficile* strains growing in BHISG under exposure to MTZ compared to non-treated controls (number of spectra).

Gene or Accession number	Description	VPI-0	VPI-MIC/4	VPI-MIC/2	146–0	146-MIC/4	146-MIC/2	Link with MTZ	Reference
*murA* VPIv1_130014	UDP-N-acetylglucosamine 1-carboxyvinyltransferase	37	18	21	25	17	18	Cell wall metabolism changes are one signature of 630Δerm stressed by MTZ	[41]
*murB* VPIv1_450118	UDP-N-acetylenolpyruvoylglucosamine reductase	0	8	0	0	6	3		
*murC* VPIv1_460104	UDP-N-acetylmuramate—L-alanine ligase	0	13	5	0	16	6		
*murD* VPIv1_401167	UDP-N-acetylmuramoylalanine—D-glutamate ligase	0	13	7	0	12	10		
*murE* VPIv1_401179	UDP-N-acetylmuramyl-tripeptide synthetase	0	4	0	0	7	1		
*murF* VPIv1_401170	UDP-N-acetylmuramoyl-tripeptide—D-alanyl-D-alanine ligase	0	10	1	0	11	5		
*murG* VPIv1_401165	N-acetylmuramyl-(pentapeptide) pyrophosphoryl-undecaprenol N-acetylglucosamine transferase	0	7	0	0	7	0		
*murI* VPIv1_470012	Glutamate racemase	0	2	0	0	3	1		
*uppS* VPIv1_400614	Undecaprenyl pyrophosphate synthetase	0	4	0	0	3	1		

**Fig 3:**
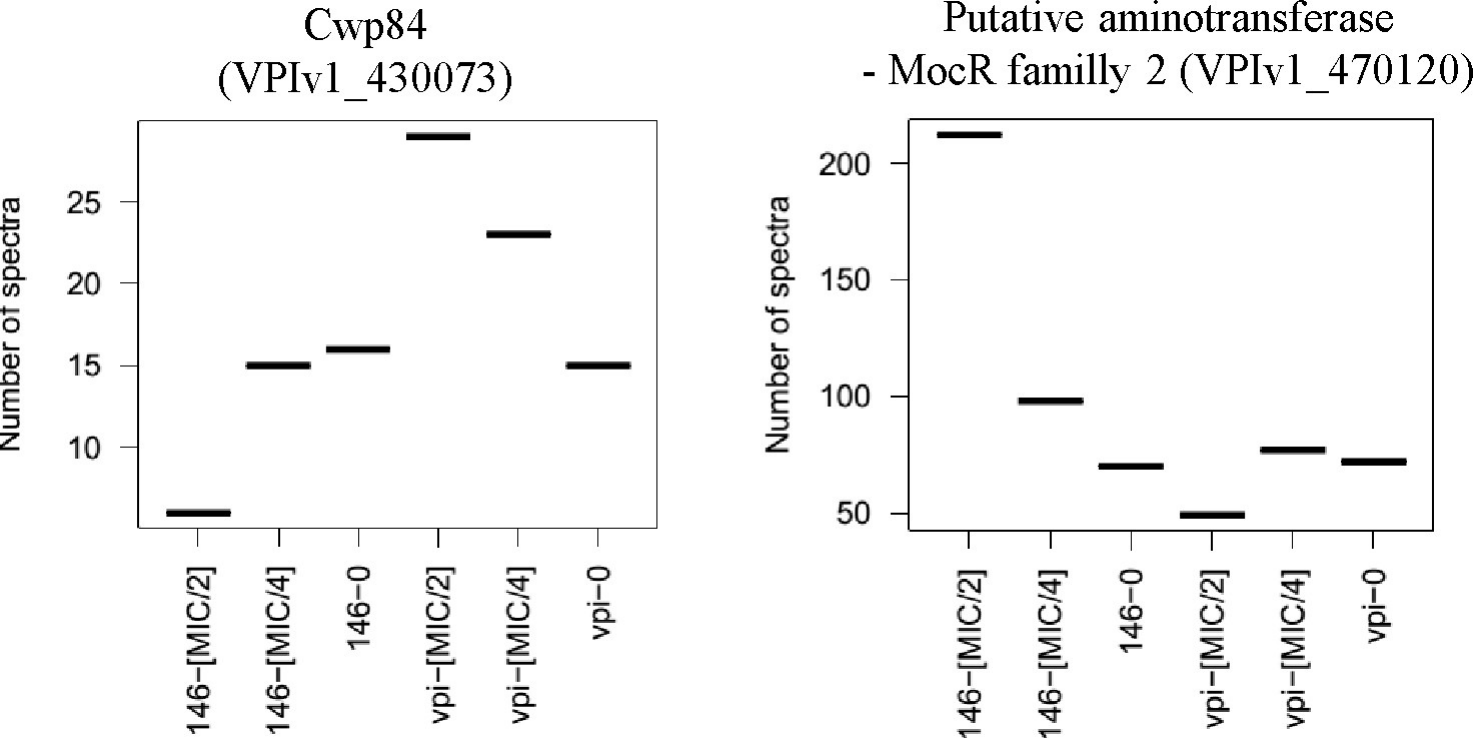
Differences in expressions of proteins involved in biofilm formation between CD17-146 and VPI 10463 strains after culture in BHISG under exposure to MTZ. The protein abundance displayed by the number of spectra that has been identified to peptides belonging to a defined protein. VPI-0 (VPI 10463, without MTZ), VPI-MIC/4 (VPI 10463, with MTZ at MIC/4), VPI-MIC/2 (VPI 10463, with MTZ at MIC/2), 146–0 (CD17-146, without MTZ), 146-MIC/4 (CD17-146, with MTZ at MIC/4), 146-MIC/2 (CD17-146, with MTZ at MIC/2). In the presence of MTZ at MIC/2, CD17-146 decreased Cwp84 production and increased an aminotransferase belonged to MocR family 2. These modifications may implicate in enhanced biofilm production.

Interestingly, we observed in the presence of MTZ at MIC/2 a 3-fold decrease in the amount of Cwp84 in CD17-146 instead of a 2-fold increase in VPI 10463 (Fig 3). Cwp84 is a protease that cleaves the S-layer protein SlpA on bacterial surface into two subunits, resulting in a significantly higher hydrophobicity on the surface. It has been shown that the *cwp84* mutant strain grew slower and elaborated biofilms with increased biomass compared with the parental *C*. *difficile* 630Δ*erm* strain [49]. In vitro and in vivo competition assays revealed that the mutant was significantly impaired for growth only in the planktonic state, but not in biofilms or in vivo. Furthermore, bacterial load of mutant strain was maintained over time in the cecum, suggesting there may be stable reservoirs of bacteria and these reservoirs may ultimately transition into the biofilm state [49].

We also observed a 3-fold increased amount of a putative aminotransferase for CD17-146 strain at MIC/2, but not for VPI 10463 (Fig 3). This protein belonging to MocR family 2 shares 27% identity with PdxR of *Streptococcus mutans*. Interestingly, PdxR is known to have a role in biofilm formation of *S*. *mutans* since the *pdxR* mutant forms significantly fewer biofilm compared to its parental strain [50]. These data (induction of PdxR and down-regulation of Cwp84) partially agree with the results of a recent study on strains PCR ribotype 010 showing that subinhibitory concentrations of MTZ increased biofilm formation in a susceptible strain and a strain with reduced susceptibility while the biofilm-forming ability of the MTZ stable-resistant strain was not further increased by the antibiotic pressure [37]. Further research is required to elucidate if MTZ can induce biofilm in CD17-146 strain.

### Impact of MTZ on electron transport and redox-active proteins

The addition of subinhibitory concentrations of MTZ led to differential production of electron transport and redox-active proteins in both VPI 10463 and CD14-146. It is well known that activation of MTZ requires a reduction under sufficiently low redox potential in anaerobic organisms [51]. Studies on *B*. *fragilis* and *H*. *pylori* revealed that several proteins involved in electron transfer reactions (i.e. pyruvate ferredoxin/flavodoxin oxidoreductase, ferredoxin, hydrogenases, etc.) are important for the reductive activation of MTZ [17,52].

**Fig 4:**
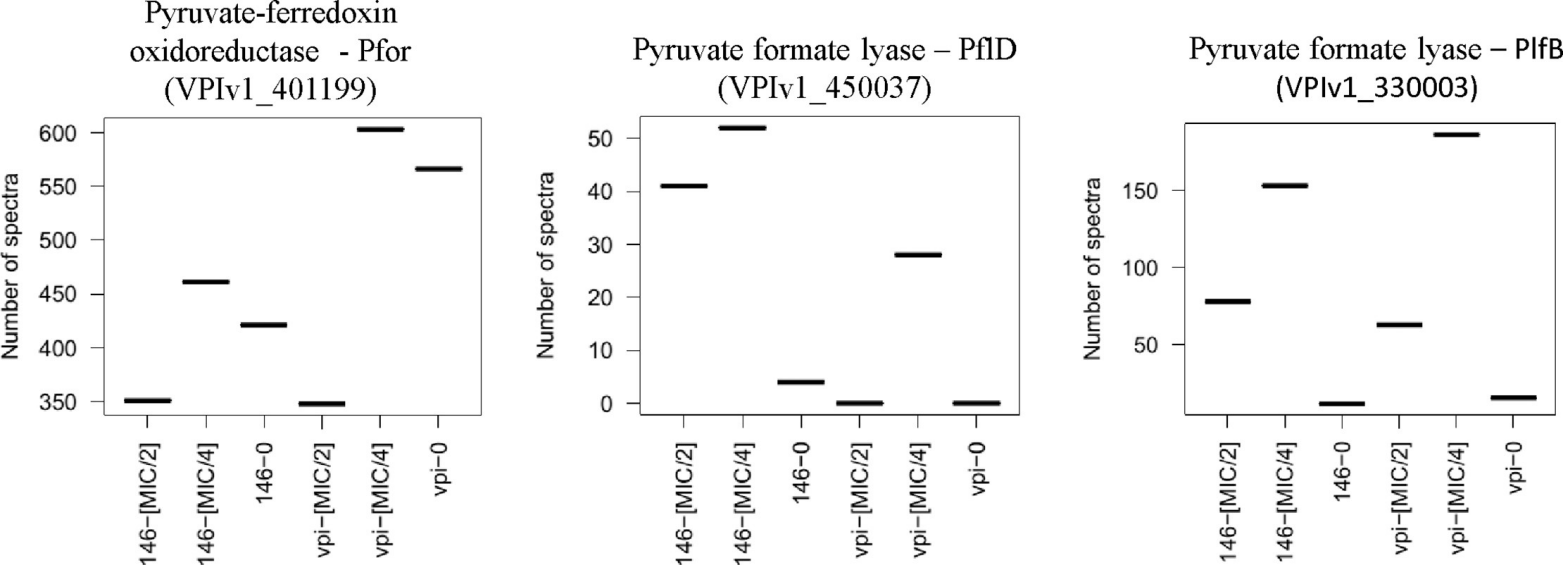
Differential production in electron transport and redox-active proteins of CD17-146 and VPI 10463 after culture in BHISG without MTZ and with MTZ. The protein abundance displayed by the number of spectra that has been identified to peptides belonging to a defined protein.VPI-0 (VPI 10463, without MTZ), VPI-MIC/4 (VPI 10463, with MTZ at MIC/4), VPI-MIC/2 (VPI 10463, with MTZ at MIC/2), 146–0 (CD17-146, without MTZ), 146-MIC/4 (CD17-146, with MTZ at MIC/4), 146-MIC/2 (CD17-146, with MTZ at MIC/2). In the presence of MTZ at MIC/2, the amount of Pfor, which activate MTZ in bacteria, decreased in both strains. To establish alternative route for pyruvate metabolism, the production of two pyruvate-formate-lyase increased.

Pyruvate ferredoxin oxidoreductase (PFOR) together with ferredoxine (Fd) catalyzes the CoA-dependent decarboxylation of pyruvate to acetyl-CoA. PFOR/Fd couple is the only one with a low enough redox potential capable of reducing MTZ in anaerobic bacteria. The prodrug MTZ is activated to its toxic, radical state by the electrons transferred to the amine group of MTZ from Fd, which is itself reduced by the key metabolic enzyme PFOR [9]. The decrease of PFOR activity is commonly associated with MTZ resistance in *H*. *pylori*, *C*. *perfringens* and *Bacteroides spp*. [11]. The amount of PFOR was 1.3-fold more important in VPI 10463 than in CD17-146 after culture without MTZ. In the presence of MTZ at MIC/2, the amount of PFOR decreased 1.6-fold and 1.2-fold for VPI 10463 and CD17-146, respectively (Fig 4), likely limiting the activation of MTZ. Furthermore, if MTZ remained unreduced, drug uptake may have dropped down since a gradient for continued intracellular diffusion would not be established [53]. These results suggest that reduction of PFOR could play a role in the protection from stress induced by MTZ in *C*. *difficile*.

To establish alternative route for pyruvate utilization, the two strains upregulated pyruvate-formate-lyase (PflD and PflB). The production of PflD in strain CD17-146 was enhanced 13- and 10-fold, respectively at MIC/4 and MIC/2, while PflD was only detected at MIC/4 in strain VPI 10463 (Fig 4). The amount of PlfB increased 13- and 7-fold for CD17-146 strain and 12- and 4-fold for VPI 10463 strain after exposure to MTZ at MIC/4 and MIC/2, respectively.

Effect of MZT addition on the production of proteins involved in protection from oxidative/nitrosative stress and thiol homeostasis.

Reductive activation of the nitro group of MTZ results in the formation of nitro free radical leading to cytotoxicity. Moreover, as oxygen has a higher affinity for electrons than MTZ, it has been shown that an electron from the nitroso-radical can be removed by oxygen and MTZ is regenerated. This “futile cycling” process generates toxic oxygen radicals that can induce DNA strand breaks. Then, in addition to direct nitrosative stress, MTZ may also indirectly accelerate the damage due to oxidative stress [40].

Our data indicated higher production of other redox-active proteins in both VPI 10463 and CD17-146 with subinhibitory concentrations of MTZ, including anaerobic nitric oxide reductase (NorV), which is associated with protection against nitrosative/oxydative stress (Table 2). The production of NorV rose 6-fold for CD17-146 at MIC/4 and MIC/2. For VPI 10463, the amount of the enzyme also rose 6- and 4-fold at MIC/4 and MIC/2, respectively. Moreover, a putative nitroreductase family protein was also synthesized 2- and 2.5-fold more at MIC/2 for VPI 10463 and CD17-146, respectively (Table 2). Nitroreductases consist of two types of enzymes: oxygen-insensitive NAD(P)H pairing (type I) and oxygen-sensitive (type II). Type I nitroreductases render MTZ inactive by reduction of nitro group to the non-toxic stable amino, while type II nitroreductases transforms MTZ to a nitro free-radical, leading to cytotoxicity [11]. Further analysis would be required to verify whether this nitroreductase family protein belongs to type I and contributes to the level of MTZ resistance.

**Table 2:** Differential expressions of proteins involved in redox chain and stress response of VPI 10463 and CD17-146 *C*. *difficile* strains growing in BHISG under exposure to MTZ compared to non-treated controls (number of spectra).

Gene or Accession number	Description	VPI-0	VPI-MIC/4	VPI-MIC/2	146–0	146-MIC/4	146-MIC/2	Link with MTZ	Reference
*norV* VPIv1_360102	Anaerobic nitric oxide reductase flavorubredoxin	9	43	35	6	41	41	Protection against oxidative stress	This study
VPIv1_360068	Nitroreductase-family protein	15	25	22	16	11	33		
VPIv1_390030	Rev Rbr, O2/H2O2 reductase	46	129	70	75	83	37		
VPIv1_370176	Rev Rbr, O2/H2O2 reductase	44	117	60	62	78	32		
*hcp* VPIv1_400648	Hydroxylamine reductase	0	2	0	2	8	7		
VPIv1_400259	Coenzyme A disulfide reductase	0	20	3	0	17	22	Maintain of thiol homeostasis under oxidative stress	
VPIv1_401048	Coenzyme A disulfide reductase	6	19	7	0	7	0		
*trxA VPIv1_400141*	Thioredoxin 1	3	3	5	3	4	2		
*trxB* VPIv1_400142	Putative thioredoxin-disulfide reductase	1	17	5	0	16	0		
*msrAB* VPIv1_400646	Peptide methionine sulfoxide reductase MsrA/MsrB	1	8	4	1	4	5		

Other proteins implicated in protection against oxidative/nitrosative stress such as reverse-rubrerythrins (RevRbr) and hydroxylamine reductase (Hcp) were also differentially produced. The two RevRbrs increased 3- fold at MIC/4 for VPI 10463. A recent study showed that RevRbrs are O_2_- and H_2_O_2_-reductases, which have a key role to the ability of *C*. *difficile* to grow in the presence of low oxygen tension [54]. On the contrary, for CD17-146, their amount did not change at MIC/4 and decreased by half at MIC/2 (Table 2). In CD17-146 strain, the Hcp protein was undetectable without MTZ and became detectable at MIC/4 and MIC/2. The increased production of Hcp was not observed in VPI 10463. These observations suggest slight differences in the response of the two strains to oxidative/nitrosative stress of two strains (Table 2). Protein thiols are targets of oxidative/nitrosative stress as well. After induction by MTZ, especially at MIC/4, we observed in both strains a higher production of a variety of proteins, which ensure that cytosolic thiol groups are maintained in their reduced state and prevent the formation of stable disulfide bonds, such as Coenzyme A disulfide reductase, thioredoxin (TrxA), thioredoxin-disulfide reductase (TrxB) and peptide methionine sulfoxide reductase (MsrAB) (Table 2).

It is worth noting that the TrxAB system, one RevRbr and NorV proteins, which are believed to be associated with protection against the effect of nitric oxide, ROS and oxygen were more produced in a stable, metronidazole-resistant *C*. *difficile* isolate [28]. Further analysis will be needed to determine the role of these proteins in the responses to MTZ.

### Impact of MTZ on DNA repair and stress response proteins

For both strains, production of proteins involved in DNA repair also increased after treatment with MTZ. One such protein is the excinuclease UvrA (UvrABC repair system), which is involved in the repair of DNA lesions. Without MTZ this enzyme was under detection threshold, while its amount strongly increased following addition of MTZ at MIC/4 (Table 3). We also observed a slight increase of other proteins implicated in DNA repair at MIC/4 such as UvrB, UvrC, UvrD, the DNA mismatch repair protein (MutS), a DNA glycosylase as well as ribonucleoside-diphosphate reductase (NrdE) and a triphosphate reductase (NrdD).

**Table 3:** Differential expressions of proteins involved in DNA repair and stress response of VPI 10463 and CD17-146 *C*. *difficile* strains growing in BHISG under exposure to MTZ compared to non-treated controls (number of spectra).

Gene or Accession number	Description	VPI-0	VPI-MIC/4	VPI-MIC/2	146–0	146-MIC/4	146-MIC/2	Link with MTZ	Reference
*uvrA* VPIv1_450127	Excinuclease ABC subunit A	0	20	8	0	23	1	Repair DNA damaged by MTZ stress	[8]
*uvrB* VPIv1_450128	Excinuclease ABC subunit B	0	4	0	0	7	0		
*uvrC* VPIv1_450126	Excinuclease ABC subunit C	0	0	0	0	2	0		
VPIv1_370019	Putative DNA helicase, UvrD/REP type	0	6	0	0	5	1		
*mutS* VPIv1_400422	DNA mismatch repair protein	0	6	0	0	5	0		
VPIv1_130123	Putative DNA glycosylase	0	4	0	0	3	0		
*nrdE* VPIv1_430224	Ribonucleoside-diphosphate reductase subunit alpha	0	5	0	0	1	0		
*nrdD* VPIv1_110002	Anaerobic ribonucleoside triphosphate reductase	0	22	15	0	24	0		
*recA* VPIv1_370016	Protein RecA (Recombinase A)	1	11	11	0	18	11		
*grpE* VPIv1_400967	Protein GrpE (HSP-70 cofactor)	5	12	16	1	14	12	Refold proteins denatured by oxidative stress	[40]
*dnaK* VPIv1_400966	Chaperone protein dnaK (Heat shock protein 70) (HSP70)	37	66	82	82	69	109		
*clpB* VPIv1_400479	Chaperone protein	18	15	30	26	17	71		
*clpC* VPIv1_50007	class III stress response-related ATPase, AAA+ superfamily	5	16	32	5	10	51		
*clpX* VPIv1_450060	ATP-dependent Clp protease ATP-binding subunit ClpX	0	11	1	0	18	1		
*htpG* VPIv1_140085	Heat shock protein 90	0	42	21	0	32	36		
*Lon* VPIv1_450056	ATP-dependent protease La, S16 peptidase family	0	23	3	0	19	3		

Another important DNA repair protein, RecA, was more produced in both strains. In the absence of MTZ, RecA was undetectable while its amount increased in the presence of MTZ and became detectable at MIC/4 and MIC/2 (Table 3). RecA is involved in ATP-dependent strand exchange reaction of DNA in homologous recombination and repair of double-stranded DNA breaks. It also plays a central role in the SOS response. RecA is thought to be part of the adaptive response to MTZ in *B*. *fragilis* and *H*. *pylori* [18,25]. In addition, several reports indicated that *E*. *coli* mutant deficient in *recA* were 10-fold more susceptible to MTZ than parental strains [11]. It would be interesting to determine if RecA also plays a role in MTZ susceptibility in *C*. *difficile*.

Various cellular stress-related proteins were also more synthesized after treatment with MTZ (Table 3). The Lon protease, which hydrolyzes misfolded proteins, was strongly upregulated at MIC/4 for both strains while it was under detection threshold without MTZ. HtpG, DnaK, GrpE and Clp proteins also function to refold denatured proteins, which may be the result of heat, oxidative, acid and osmotic/ionic stress [55]. HtpG was undetectable in the absence of MTZ. Its production increased for VPI 10463 and CD17-146 at MIC/4 and MIC/2. Meanwhile, in the presence of MTZ at MIC/2, for VPI 10463 and CD17-146 respectively, DnaK production increased 2- and 1.5-fold, ClpB increased 1.6- and 3-fold, ClpC synthesis increased 6- and 8-fold and GrpE increased 4- and 10-fold. It seems that CD17-146 induced more strongly the heat-stress related proteins in the presence of MTZ, especially at MIC/2.

### Impact of MTZ on protein biosynthesis

In the presence of subinhibitory concentration of MTZ, it was worth mentioning changes in synthesis of proteins involved in translation pointing to a substantial reprogramming of protein production. Indeed, the amounts of 3 elongation factors (EF-G, EF-Ts, EF-Tu) and several tRNA synthetases (AlaS, ArgS, AspS, CysS, GlnS, GltX, GlyQ, GlyS, IleS, LeuS, LysS, MetG, PheT, ProS, SerS, ThrS, TyrS, ValS) were found to drastically increase in both strains in the presence of MTZ at MIC/4. Conversely, their production decreased at MIC/2 compared to MIC/4 (Table 4). These changes have been previously shown in a study on proteomic signatures of *C*. *difficile* stressed with sub lethal concentrations of MTZ, vancomycin, or fidaxomicin in exponential growth phase [56]. In addition to their roles in translation, some aminoacyl-tRNAs are also used in pathways directly implicated in antibiotic resistance. Aminoacylation of membrane lipids adjusts the negative charge of the membrane bilayer and controls the membrane permeability to cationic antimicrobials. This process is dependent on the aminoacyl-tRNA substrate [57].

**Table 4:** Differential expression of protein involved in protein biosynthesis and amino acid metabolism of VPI 10463 and CD17-146 *C*. *difficile* strains growing in BHI-SG under exposure to MTZ compared to non-treated controls (number of spectra).

Gene	Description	VPI-0	VPI-MIC/4	VPI-MIC/2	146–0	146-MIC/4	146-MIC/2	Link with MTZ	Reference
** **	**Protein biosynthesis**	** **						** **	** **
*fusA* VPIv1_80013	Elongation factor G (EF-G)	8	129	36	8	169	89	Increase of protein biosynthesis is one signature of 630Δ*erm* stressed by MTZ	[41]
*tuf* VPIv1_70001	Elongation factor EFTu/EF1A	16	147	34	13	209	58		
*tsf* VPIv1_400618	Elongation factor Ts (EF-Ts)	20	49	47	9	51	29		
*alaS* VPIv1_360288	Alanyl-tRNA synthetase	0	33	4	0	36	19		
*argS* VPIv1_190133	Arginyl-tRNA synthetase (ArgRS)	0	23	7	0	33	18		
*asnC* VPIv1_400734	Asparaginyl-tRNA synthetase (AsnRS)	28	27	28	25	30	23		
*aspS* VPIv1_430023	Aspartyl-tRNA synthetase (AspRS)	2	38	13	2	35	25		
*cysS* VPIv1_60023	Cysteinyl-tRNA synthetase (CysRS)	0	42	15	0	39	22		
*hisS* VPIv1_430024	Histidyl-tRNA synthetase (HisRS)	3	9	5	6	10	10		
*glnS* VPIv1_400526	Glutaminyl-tRNA synthetase	0	30	1	0	27	9		
*gltX* VPIv1_60021	Glutamyl-tRNA synthetase	0	29	3	0	33	18		
*glyQ* VPIv1_400935	Glycyl-tRNA synthetase alpha subunit (GlyRS)	0	19	6	1	16	14		
*glyS* VPIv1_400934	Glycyl-tRNA synthetase beta subunit (GlyRS)	0	41	12	0	38	22		
*ileS* VPIv1_401127	Isoleucyl-tRNA synthetase	0	85	21	7	91	68		
*leuS* VPIv1_401028	Leucyl-tRNA synthetase	0	46	4	2	55	24		
*lysS* VPIv1_470002	Lysyl-tRNA synthetase (LysRS)	4	35	19	1	32	20		
*metG* VPIv1_460125	Methionyl-tRNA synthetase (MetRS)	0	46	6	1	42	19		
*pheT* VPIv1_190122	Phenylalanyl-tRNA synthetase subunit beta (PheRS)	0	42	10	1	37	33		
*pheS* VPIv1_190121	Phenylalanyl-tRNA synthetase alpha chain (PheRS)	0	18	2	0	18	14		
*proS* VPIv1_60020	Prolyl-tRNA synthetase	7	56	21	27	74	55		
*serS* VPIv1_20003	Seryl-tRNA synthetase 1	1	25	7	7	33	28		
*selB* VPIv1_400998	Selenocysteinyl-tRNA-specific translation factor	0	11	1	0	15	4		
*thrS* VPIv1_180093	Threonyl-tRNA synthetase	3	50	17	1	48	37		
*tyrS* VPIv1_390027	Tyrosyl-tRNA synthetase (TyrRS)	2	25	17	5	21	23		
*valS* VPIv1_430566	valyl-tRNA synthetase	2	38	15	9	37	29		
* *	**Amino acid metabolism**								
*ldhA* VPIv1_160032	D-lactate dehydrogenase	10	37	20	12	60	84	Adaptive metabolism of *C*. *difficile* when ATP recoveries is very low, suggesting MTZ had an impact on ATP sources	[43]
*hadI* VPIv1_160034	Activator of 2-hydroxyisocaproyl-CoA dehydratase	2	14	5	2	16	14		
*hadB* VPIv1_160035	Subunit of oxygen-sensitive 2-hydroxyisocaproyl-CoA dehydratase B	20	113	20	76	95	120		
*acdB* VPIv1_160037	Acyl-CoA dehydrogenase, short-chain specific	32	141	47	56	140	144		
*rnfC* VPIv1_360081	Electron transport complex protein	0	8	4	0	7	1		
*rnfB* VPIv1_360086	Electron transport complex protein	0	2	1	0	0	3		

### Effect of MTZ on the synthesis of enzymes involved in amino acid metabolism

Several enzymes involved in reductive Stickland metabolism (mainly Leucine catabolism) were more produced at MIC/4 for VPI 10463 and at both MIC/4 and MIC/2 for CD17-146 (LdhA, HadI, HadB, AcdB, and RnfC) (Table 4). At MIC/4 in VPI 10463 strain, LdhA production increased 4-fold, HadI increased from 2 to 14 spectra, HadB synthesis increased 6-fold and AdcB increased 4-fold, while at MIC/2, only LdhA increased 2-fold. For CD17-146 strain, at MIC/4 and MIC/2 respectively, LdhA increased 5- and 7-fold, HadI increased 8- and 7-fold, HadB increased slightly 1.3- and 1.6-fold, AcdB increased 2.5-fold at both concentrations. Moreover, RnfC was not detected in the absence of MTZ and became detectable at MIC for both strains at MIC/4. In Stickland metabolism, *C*. *difficile* combined amino acid fermentation via electron bifurcation to membrane potential generating processes at the Rnf complex that couples the NADH-dependent reduction of enoyl-CoA obtained from amino acid catabolism to the reduction of ferredoxin. Two electrons derived from NADH are distributed to two acceptors, enoyl-CoA and ferredoxin. The free energy resulting from redox potential difference between ferredoxin and NAD^+^ is used to transport ions across the membrane and to generate ATP. This reductive Stickland metabolism is known to be the highly adaptive metabolism of *C*. *difficile* when normal substrate level phosphorylation suffers from a deficiency of ATP [58]. The higher production of proteins implicated in the pathway suggests a lack of energy in *C*. *difficile* in the presence of MTZ.

### Impact of MTZ on carbohydrate metabolism

**Fig 5:**
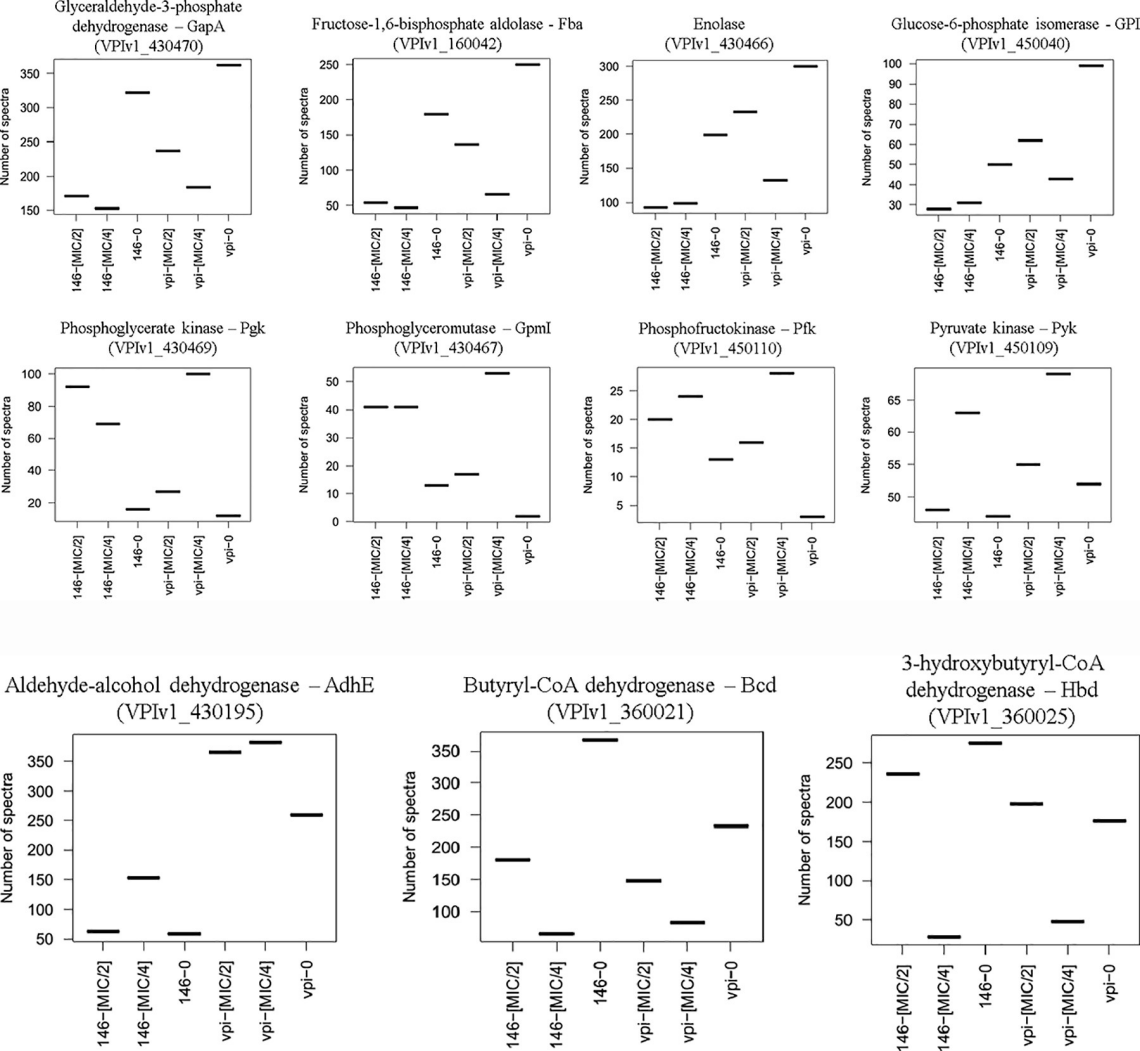
Modification in the amount of proteins involved in glycolysis for CD17-146 and VPI 10463 after culture in BHISG with subinhibitory concentrations of MTZ. **A.** The protein abundance displayed by the number of spectra that has been identified to peptides belonging to a defined protein.VPI-0 (VPI 10463, without MTZ), VPI-MIC/4 (VPI 10463, with MTZ at MIC/4), VPI-MIC/2 (VPI 10463, with MTZ at MIC/2), 146–0 (CD17-146, without MTZ), 146-MIC/4 (CD17-146, with MTZ at MIC/4), 146-MIC/2 (CD17-146, with MTZ at MIC/2). The amount of several enzymes involved in glycolysis decreased in the presence of MTZ, except for some enzymes involved in substrate phosphorylation which were overproduced. **B.** Modification in the amount of proteins involved in fermentation for CD17-146 and VPI 10463 after culture in BHISG with subinhibitory concentrations of MTZ. The protein abundance displayed by the number of spectra that has been identified to peptides belonging to a defined protein.VPI-0 (VPI 10463, without MTZ), VPI-MIC/4 (VPI 10463, with MTZ at MIC/4), VPI-MIC/2 (VPI 10463, with MTZ at MIC/2), 146–0 (CD17-146, without MTZ), 146-MIC/4 (CD17-146, with MTZ at MIC/4), 146-MIC/2 (CD17-146, with MTZ at MIC/2). Differences in the production of proteins involved in fermentation pathways were also observed in the presence of MTZ: increase of AdhE and diminution of Bcd and Hbd.

Additionally, it should be noted that the amount of several proteins involved in glycolysis were modified after MTZ stress in both strains. The quantity of several enzymes decreased about 2-fold in the presence of MTZ: glucose-6-phosphate isomerase (Pgi), glyceraldehyde-3-phosphate dehydrogenase (GapA), fructose-1,6-bisphosphate aldolase (Fba), enolase (Eno) (Fig 5A). The drop in glucose metabolism might contribute to reduced susceptibility of bacteria by diminution of energy-dependent uptake of MTZ. Indeed, it has been described that the uptake of MTZ by an energy-dependent mechanism in *Clostridium pasteurianum* is significantly inhibited by a number of inhibitors of glycolysis (sodium fluoride, arsenate and iodoacetic acid) [9].

Conversely, some enzymes of glycolysis involved in substrate phosphorylation were upregulated under the same conditions: the phosphoglycerate kinase (Pgk), phosphoglyceromutase (GpmI), phosphofructokinase (Pfk) and pyruvate kinase (Pyk) (Fig 5A). For CD17-146, Pfk rose about 2-fold, Pgk rose around 5 to 6-fold, GmpI rose around 3-fold at both concentrations of MTZ. For VPI 10463, at MIC/4 and MIC/2 respectively, the amount of Pfk rose 10 or 6-fold, that of Pgk rose 8- and 2-fold, GmpI rose from 2 to 53 and 17 spectra. Besides, Pyk rose slightly 1.3-fold at MIC/4 for both strains. Contrary to other enzyme of glycolysis, the production of these enzymes might be induced by the lack of energy in the cell due to a drop in glycolysis. Interestingly, it has been shown that the activity of phosphofructokinase and pyruvate kinase from *Bacillus stearothermophilus* were induced by ADP and AMP, respectively when cell energy (ATP) went down [59,60]. Our results on the modification of production of enzymes of the glycolysis in the presence of MTZ suggest that the decreased glycolysis could involve in a declined energy-dependent uptake of the antibiotic in bacterial cell.

Besides, differences in the production of proteins involved in fermentation pathways were also observed. The amount of AdhE, an aldehyde-alcohol dehydrogenase participating in ethanol and butanol production, went up 1.4-fold at both concentrations of MTZ for VPI 10463, while for CD17-146, it went up 2.5-fold only at MIC/4. Meanwhile butyryl-CoA dehydrogenase (Bcd*)* and 3-hydroxybutyryl-CoA dehydrogenase (Hbd) involved in butyrate production decreased for both strains, especially at MIC/4: Bcd amount decreased 3- and 6-fold, the amount of Hbd decreased 4- and 10-fold for VPI 10463 and CD17-146, respectively (Fig 5B).

**Fig 6:**
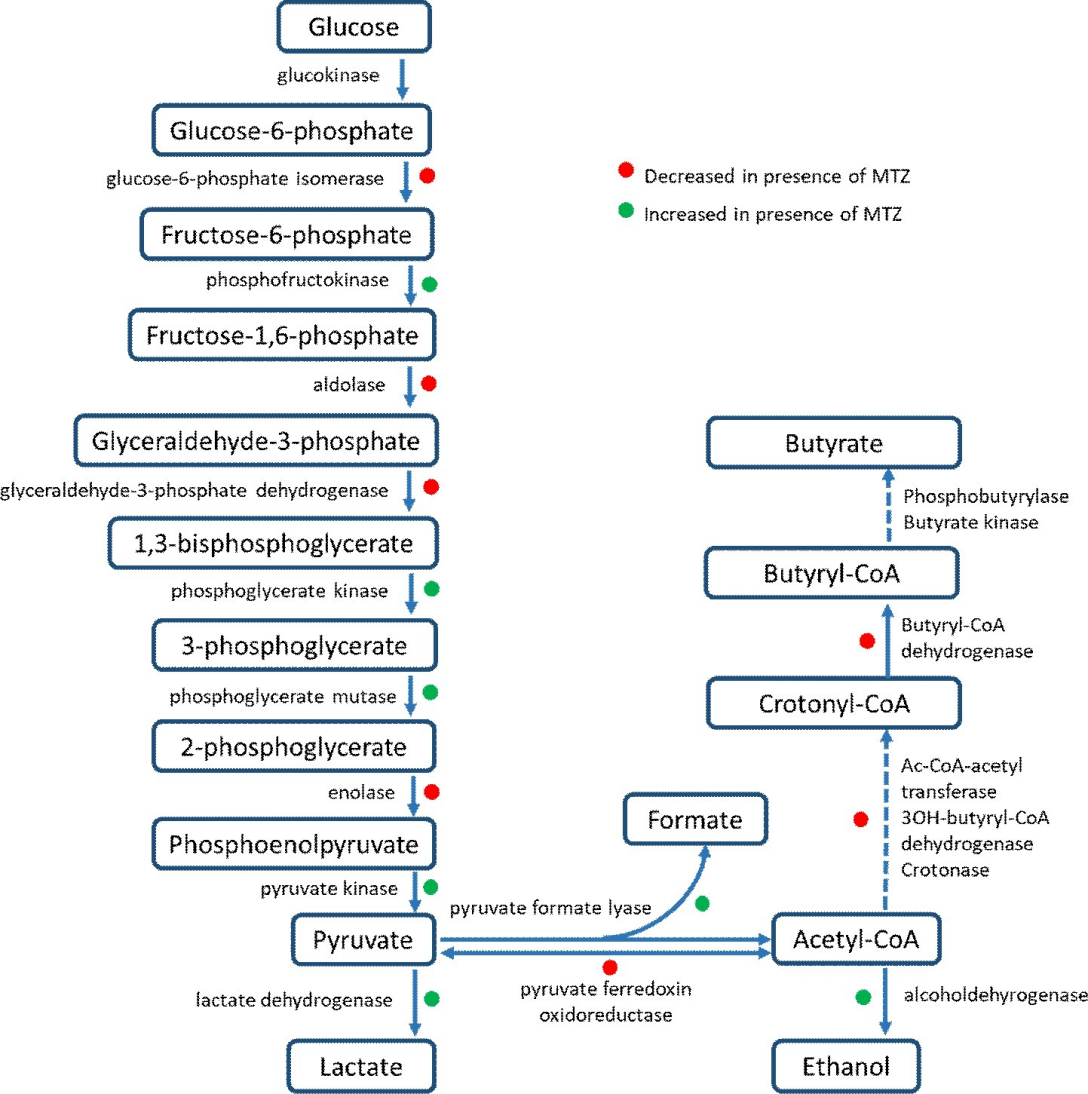
Schematic overview of the glycolysis showing the changes on glycolysis of CD17-146 and VPI 10463 after culture in BHISG with subinhibitory concentrations of MTZ. The increase or decrease in production of proteins were marked by dot green or red, respectively.

All modifications of proteins involved in carbohydrate metabolism were resumed in Fig 6.

### Role of SigB targets in the response to MTZ

SigB is known to control the expression of about 25% of genes involved in the management of various stresses, metabolism, cell surface biogenesis and sporulation and plays a crucial role in adaptive strategy of *C*. *difficile*. The *sigB* mutant has an increased sensitivity to cationic antimicrobial peptides, nitric oxide and reactive oxygen species [40]. Interestingly, we observed that several proteins found as more produced in the presence of MTZ in our study are positively controlled by SigB [40]. This includes most of the proteins involved in oxidative/nitrosative stress (NorV, RevRbr, Hcp, TrxAB, MsrAB), a nitroreductase (CD1125 in strain 630) as well as DNA repair proteins (UvrABCD, MutS, NrdE). Several genes involved in central metabolism are also differently expressed in the *sigB* mutant [40]. However, Mur enzymes negatively controlled by SigB were also induced in the presence of MTZ. We wonder if there is a link between the reprogramming of proteins synthesis following exposure to MTZ and SigB in *C*. *difficile*. Since the *sigB* mutant of the 630Δ*erm* strain is available [35], we performed a disk diffusion test (disk containing 4 μg of MTZ) on this mutant and the wild-type strain. We observed a difference between the two strains: 27.2 ±-1.4 mm zone of inhibition for 630Δ*erm* and 33.6 ±-1.6 mm for the *sigB* mutant. Taken together, this suggests that SigB could play a direct or indirect role in adaptive strategies of *C*. *difficile* in the presence of MTZ.

**Fig 7:**
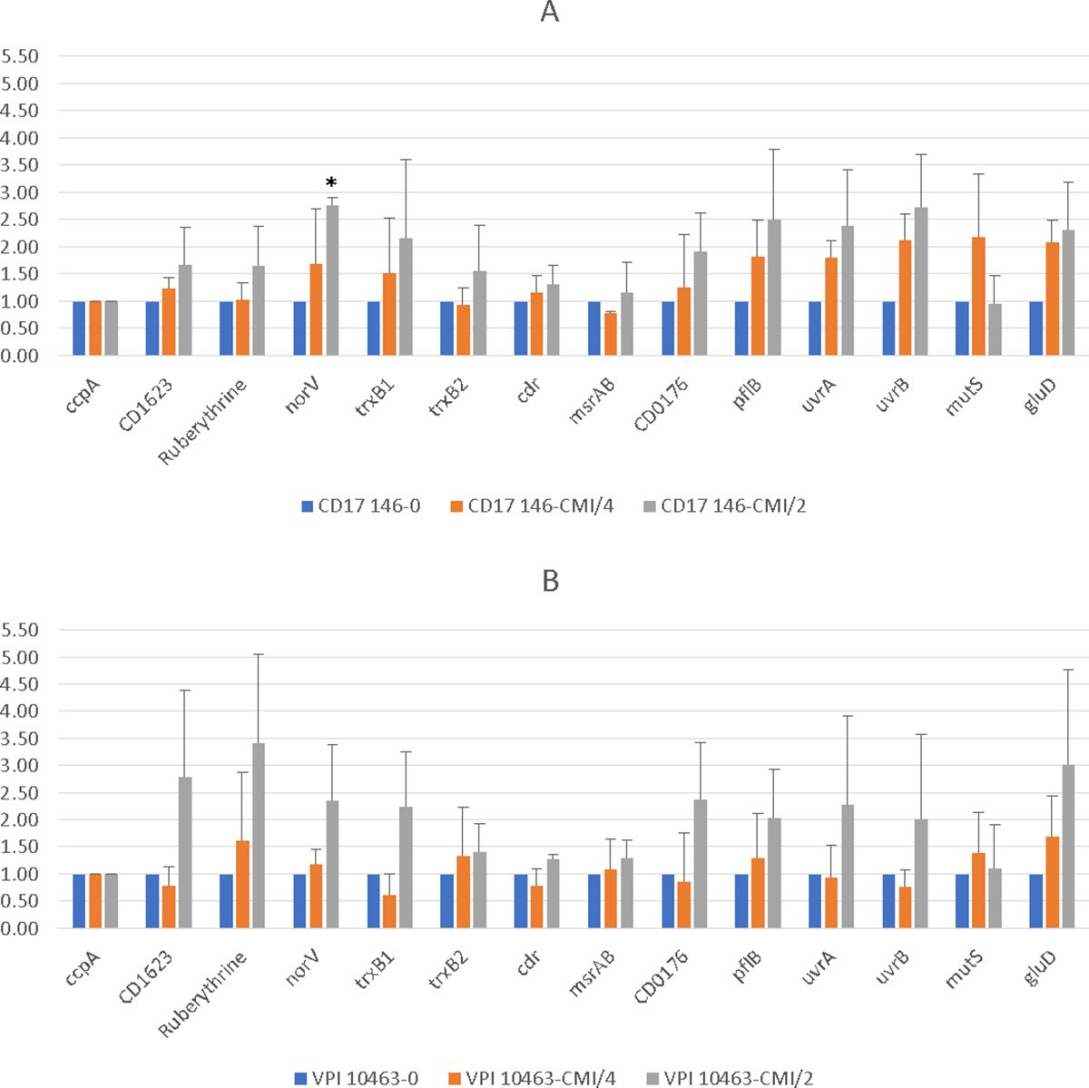
Differential expressions of SigB target genes involved in oxidative/nitrosative stress and DNA repair in CD17-146 and VPI 10463 strains after culture in BHI-SG with subinhibitory concentrations of MTZ. Fold change in expression of genes of CD17-146 (A) and VPI 10463 (B) in culture with MTZ at MIC/4 (orange) and MIC/2 (grey) compared to culture without MTZ (blue). Ruberythrine in this chart represents two copies of revRbr because the primers for q-RT-PCR cannot distinguish them. Error bars represent standard deviation. Significantly different (p<0.05) ratios are indicated by asterisks (Man-Whitney test). Data are representative of three independent experiments, each performed in duplicate. We found that *norV*, *pflB*, *trxB1*, *gluD* and *uvrAB* genes were up-regulated around 2-3-fold for both strains in the presence of MTZ.

We then studied the expression of some SigB-target genes in the two strains grown in the presence or absence of subinhibitory concentrations of MTZ. We tested some redox proteins involved in oxidative stress and DNA repair. We found that *norV*, *pflB*, *trxB1*, *gluD* and *uvrAB* genes were up-regulated around 2-3-fold for both strains in the presence of MTZ. For VPI 10463, the expression of genes encoding *revRbrs* was also upregulated more than 2-fold (Fig 7). The differences were statistically different only for the increase of *norV* expression at MIC/2 for the strain CD17-146.

Further work is needed to confirm a possible role of SigB in the response to stress induced by MTZ and the mechanism involved. However, it is interesting to note that several members of the SigB -regulon [40] are also more produced in a mutant resistant to MTZ [28].

## Conclusion

In accordance with studies of response of other anaerobes to MTZ, our results confirm that the protection of *C*. *difficil*e from stress induced by MTZ is multifactorial. Further studies are required to characterize more precisely the role of these proteins and to confirm a possible link between the SigB regulon and the response to MTZ. Even if SigB has been shown to play a role in β-lactams resistance in *Staphylococcus aureus* [61], this is the first study indicating a possible involvement of SigB in response to stress induced by MTZ.

Moreover, although both strains have similar protein profiles in absence of MTZ, their proteomes tend to diverge when exposed to subinhibitory concentrations of MTZ, indicating a heterogeneity in the ways to respond to this antibiotic stress. CD17-146 might promote biofilm production by regulation of Cwp84, a cell wall protein, and of a MocR family aminotransferase, which was not observed in VPI 10463. It is worth noting that subinhibitory concentrations of MTZ are able to enhance biofilm formation of reduced sensitivity *C*. *difficile* strains but the mechanism remains unknown [37]. To our knowledge, this work is the first description of an effect of MTZ on the production of proteins involved in biofilm formation. These proteins could be targets for further studies elucidating the mechanism of MTZ induced biofilm in *C*. *difficile*.

In addition, these two strains may use different redox-active proteins for protection against oxidative/nitrosative stress induced by MTZ. CD17-146 increased the production of a hydroxylamine reductase, Hcp while VPI 10463 upregulated RevRbr. Some pathways in amino acid degradation and glycolysis were also differently affected by MTZ. We observed that reductive Stickland metabolism was induced, and glycolysis was repressed at both MIC/4 and MIC/2 for CD17-146 suggesting a switch in energy metabolism. For VPI 10463, MTZ affects reductive Stickland metabolism and glycolysis at MIC/4 but much less at MIC/2. These results suggest that despite having similar proteins profiles in cultures without MTZ, the two strains can react in different way to stress induced by MTZ. Therefore, the reduced susceptibility may be the consequence of changes in the bacterial physiology. Recent studies have shown that continued growth in the presence of sub-MIC antibiotic levels is a crucial aspect of the current antibiotic resistance crisis as low antibiotic concentrations can generate genetic and phenotypic variability by increasing the rate of adaptive evolution [38]. Further investigations will be necessary to shed more light on the different aspects of this complex crosstalk between bacterial metabolism and the antibiotic susceptibility.

## Supporting information

S1 Table Sequences of oligonucleotide primers used in this study. (XLSX)
